# Prevalence of cognitive impairment in individuals aged over 65 in an urban area: DERIVA study

**DOI:** 10.1186/1471-2377-11-147

**Published:** 2011-11-17

**Authors:** Emiliano Rodríguez-Sánchez, Sara Mora-Simón, María C Patino-Alonso, Ricardo García-García, Alfonso Escribano-Hernández, Luis García-Ortiz, Ma Victoria Perea- Bartolomé, Manuel A Gómez-Marcos

**Affiliations:** 1 Primary care research unit of La Alamedilla Health Center, Castilla y León Health Service- SACYL, Salamanca, Spain; 2 Department of Basic Psychology, Psychobiology and Behavioral Sciences Methodology. Faculty of Psychology. University of Salamanca. Spain; 3 Department of Statistics. Faculty of Medicine. University of Salamanca. Spain

## Abstract

**Background:**

Few data are available on the prevalence of cognitive impairment (CI) in Spain, and the existing information shows important variations depending on the geographical setting and the methodology employed. The aim of this study was to determine the prevalence of CI in individuals aged over 65 in an urban area, and to analyze its associated risk factors.

**Methods:**

Design: A descriptive, cross-sectional, home questionnaire-based study; Setting: Populational, urban setting. Participants: The reference population comprised over-65s living in the city of Salamanca (Spain) in 2009. Randomized sampling stratified according to health district was carried out, and a total of 480 people were selected. In all, 327 patients were interviewed (68.10%), with a mean age of 76.35 years (SD: 7.33). Women accounted for 64.5% of the total. Measurements: A home health questionnaire was used to obtain the following data: age, sex, educational level, family structure, morbidity and functionality. All participants completed a neuropsychological test battery. The prevalence data were compared with those of the European population, with direct adjustment for age and sex. Diagnoses were divided into three general categories: normal cognitive function, cognitive impairment - no dementia (CIND), and dementia.

**Results:**

The prevalence of CI among these over-65s was 19% (14.7% CIND and 4.3% dementia). The age-and sex-adjusted global prevalence of CI was 14.9%. CI increased with age (p < 0.001) and decreased with increasing educational level (p < 0.001). Significant risk factors were found with the multivariate analyses: age (OR = 1.08, 95%CI: 1.03-1.12), anxiety-depression (OR = 3.47, 95%CI: 1.61-7.51) and diabetes (OR = 2.07, 95%CI: 1.02-4.18). In turn, years of education was found to be a protective factor (OR = 0.79, 95%CI: 0.70-0.90). Although CI was more frequent among women and in people living without a partner, these characteristics were not significantly associated with CI risk.

**Conclusions:**

The observed raw prevalence of CI was 19% (14.9% after adjusting for age and sex). Older age and the presence of diabetes and anxiety-depression increased the risk of CI, while higher educational level reduced the risk.

## Background

The prevalence of neurodegenerative diseases increases with age [1,2]. Considering that the Spanish population is among the oldest in the world (particularly the Autonomous Region of Castilla y León, where 22.5% of the inhabitants are aged over 65) [3], a substantial increase in the prevalence of cognitive impairment (CI) is to be expected in the coming years.

It is difficult to estimate prevalence figures for CI, since the diagnostic criteria are imprecise [4,5]. Indeed, the published dementia prevalence data for both Spain [2,6-8] and other European countries [1,9,10] show great variation. Therefore, it is not possible at present to provide reliable figures applicable to our setting. Although direct age-adjusted comparisons have been made among the different study populations, there are other influencing variables with greater adjustment problems, such as the setting (rural-urban), living in the home or in institutions, the diagnostic criteria used [11], or educational level. Also, more recent attempts to reformulate the constructs propose a clinical spectrum of CI ranging from mild cognitive impairment through dementia and a corresponding physio-pathological substrate believed to be responsible for the clinical symptoms [4,5]. The analysis of interventions that may prove effective in preventing the problems associated with CI is generating much interest, since the established therapies applied to CI are scarcely effective. However, the data available on the prevalence of CI in Spain are even more limited than in the case of dementia, and show important variations depending on the geographical setting and the methodology employed.

The present study carry out to estimate the prevalence of CI in the urban population over 65 years of age in the city of Salamanca (Spain), and to describe the factors associated with CI.

## Methods

### Study design

An observational, descriptive, cross-sectional population study.

### Setting

The reference population was that of the city of Salamanca, with 172,375 inhabitants, of which 19.74% (34,020) were aged over 65. It includes 10 healthcare areas, each with a population of between 9,000 and 26,000 inhabitants.

### Participants

We selected all those aged over 65 on 1 January 2009 and living in the city of Salamanca (urban setting). A door-to-door population-based survey was carried out during the months of May to November 2009. Two weeks before the interviews, letters were sent to the selected individuals, explaining the purpose of the study and requesting their cooperation. Confidentiality of data was guaranteed. Ten days after sending the letters, a telephone call was made to arrange a home interview.

### The following exclusion criteria were applied

1) deceased individuals; 2) errors in address: a) when the letter was returned, or when the selected person or some reliable informer could not be located after 4 visits to the home or 4 telephone calls on different dates and at different times; b) persons who had moved out of the study area; and 3) those individuals who declined to participate in the study.

### Ethical aspects

The protocol was approved by the Research Ethics Committee of Salamanca University Hospital. Participants signed the consent document after receiving the first explanatory letter providing information on the study.

### Training of the evaluators

The principal researcher coordinated the entire process. The evaluators were four psychologists trained by one of the researchers (SMS) to carry out the interview with the programmed questionnaires. A manual was drafted, describing the appointment procedure and application of the interviews, and was reviewed with the interviewers over two sessions. We also used two recorded home interviews in the training sessions. During the study, communication was permanently maintained for resolving any doubts or dealing with incidents in relation to the questionnaires.

### Data sources

The sample was taken from the Castilla y León Regional Health Service lists, which cover 99.5% of the population. The lists included both community dwellers and institutionalized elders.

### Study size

**Figure 1:**
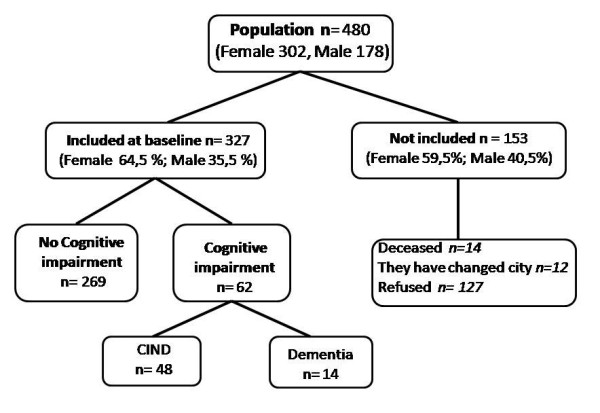
**Flow chart of the DERIVA Study**.

Accepting an alpha risk of 0.05 and a beta risk of 0.20, estimating a CI prevalence of about 16% [12], with an error of 4%, and considering the current population aged over 65, a total of 320 patients was required. Assuming a loss rate of up to 50% due to non-responses, as observed in similar studies, the calculated sample size was 480 individuals. In the secondary analysis of cases and controls, with a 327 participants sample, the statistical power was of 79.4% to detect an Odds Ratio of 2.5 with a confidence level of 95% (Epidat 4.0). We carried out a stratified random sampling by health districts. In order to reach the required sample size, we made a replacement for lost participants. The sample size of each health district was proportional to its population over 65 years. In a first stage, 260 interviews were carried out, accounting for 80% of the required sample. Two months later, in a second stage, we replaced the losses within each health district and 67 more people were interviewed. The most common cause of losses was patient refusal to participate (83.0%). This was particularly the case among the younger individuals (mean age: 75.94 ± 7.01 years) (p < 0.001). In turn, 12 patients were excluded because they had moved out of the study district (7.8%), while 14 had died (9.2%) (Figure 1). There were no significant differences between sexes regarding the cause of losses, or between losses in the first and second recruitment stages. Non-responders represented 34.83% of the males and 30.13% of the females. The distribution by age groups is shown in Table 1. Mean age of the males was 76.61 years (SD: ± 7.65), versus 77.52 years (SD: ± 7.92) in the females. There were no differences in distribution between the different sex and age categories. A total of 327 participants were interviewed, representing 68.1% of those selected (Figure 1). Of these, 116 were men (35.5%) and 211 were women (64.5%), with a mean age of 76.35 years (SD:± 7.33) similar in the two sexes.

**Table 1:** Distribution according to gender and different age ranks of interviewed and lost sample population.

	Female (N = 302)		Male (N = 178)		Total (N = 480)	
**Age (years)**	**Interviewed**	**Lost**	**Interviewed**	**Lost**	**Interviewed**	**Lost**

Mean (SD)	76.64 (7.64)	77.52(7.08)	75.81 (6.73)	76.61 (7.65)	76.35 (7.33)	77.15 (7.80)

65-69 (%)	21.3	17.6	20.74	19.4	21.1	18.3

70-74 (%)	21.8	20.9	22.4	25.8	22.0	22.9

75-79 (%)	20.4	25.3	27.6	21.0	22.9	23.5

80-84 (%)	19.5	17.6	19.0	14.5	19.3	16.3

≥ 85 (%)	17.1	17.1	10.3	19.3	14.7	19.0

Total: N (%)	211 (64.5%)	91 (59.5%)	116 (35.5%)	62 (40.5%)	327 (100%)	153 (100%)

### Measurements

A health questionnaire was administered (OARS Multidimensional Functional Assessment Questionnaire) [13] in the participant's home, for obtaining data on sociodemographics (age, sex, marital status), years of education, basic and instrumental activities of daily living, personal situation (people living with the patient, number of living offspring), morbidity (Charlson comorbidity index [14]), drug use and neuropsychological aspects. Marital status was classified according to whether the person was living with or without a partner (widowed, single, separated), while educational level was classified as follows: illiterate (failure to complete basic education), primary-secondary education (4-9 years of education) and higher education (over 9 years). Regular professional occupation before retirement was defined as domestic chores, full-time job, others (part-time job, long-term unemployment, etc.). The Katz Index of Independence in Activities of Daily Living (Katz ADL) was applied to assess functional status as a measure of patient ability to perform activities of daily living (ADLs) independently [15]. This test provides information on dependence or independence, not only in terms of the number of areas, but also identifying the specific areas. The information obtained is of a qualitative and descriptive nature, and does not provide a total score on the person's functional state.

At the beginning of the interview, neuropsychological assessment of patient cognitive status was carried out using a brief neuropsychological test battery including the following: Mini-Mental State Examination (MMSE) [16] in its validated Spanish version [17] to evaluate general cognitive state, with the possibility of assessing cognitive functions separately; the 7 Minute Screen [18] in its validated Spanish version [19] also to evaluate general cognitive state, as well as temporal orientation, memory, constructive praxias and language, separately; the Benton temporal orientation test [20] to evaluate temporal orientation; the Enhanced cued recall test [21] to evaluate episodic memory; the Clock drawing test [22] to evaluate constructive praxias; and the Categorical fluency task [23] to evaluate language. The cut-off points for cognitive impairment established for each test are as follows: MMSE<24; 7MS≤ percentile 20; Benton temporal orientation test ≤ 102; Enhanced cued recall test ≤ 12; Clock drawing test <3; Verbal fluency ≤ 10.

During the structured interview, participants were asked to present whatever relevant clinical records they might have, together with details of their current medications. In the 16 patients who were unable to complete the neuropsychological evaluation because of a deteriorated clinical condition - heart failure (2 cases), deafness (4 cases), severely impaired vision (2 cases) or severe mental impairment (4 cases) - the clinical and objective data were collected from the presented documentation, or by interviewing the caregiver or informant who knew the individual. We considered as reliable informants, in order of preference, a family member living in the same house as the individual (spouse, son/daughter, sibling); a person responsible for the care of the individual; someone living in the same house but not a family member; or a relative of the person not living in the same house. At the end of the interview the interviewers drafted a report on the quality of the information collected and on the social and health conditions of the person interviewed. Seventeen participants lived in homes for the elderly (5.2%). All the information was evaluated by the researchers (ERS, SMS, RGG and MVP) with a view to establishing the final diagnosis. The diagnoses were divided into three general categories: normal cognitive function, cognitive impairment - no dementia (CIND), and dementia.

### Classified as cognitive impairment - no dementia (CIND)

CIND was defined as: 1) mild cognitive or functional impairment reported by the participant or informant that did not meet criteria for dementia; or 2) performance on neuropsychological or functional measures that was both below expectations and ≥ 0.5 standard deviations below published norms on any test [24].

### Classified as dementia

A diagnosis of dementia was made based on the Diagnostic and Statistical Manual of Mental Disorders (IV Edition) criteria: the participant must present the development of multiple cognitive deficits including memory impairment and impairment in at least one other cognitive domain representing a decline from the previous level of functioning and of sufficient severity to cause impairment in function [4,5]. At a functional level, the person must present dependence in at least two functional areas, leading to interference in basic activities of daily living. Alterations at both the cognitive and functional levels were indicated by low performance and scores below the cut-off points in the neuropsychological and functional tests. As regards functional state, the person must present a minimum level of alteration in at least two functional areas. All of this must be accompanied by concern on the part of the participants about a change at a cognitive level compared to his or her previous state [4,5,25].

### Statistical analyses

The raw prevalence of CIND and dementia were calculated taking into account the total cases of CIND and dementia with respect to the total study sample. We estimated both global prevalence and specific prevalence per age group, sex, educational level, morbidity and functionality. Calculation was made of the 95% confidence intervals (95%CI) for the global and specific prevalence, together with prevalence adjusted for age and sex, using the European standard population [26] as a basis for adjustment (weighting: 36.4, 27.3, 18.2, 9.1 and 9.1 for age intervals of 65-69, 70-74, 75-79, 80-84 and = 85 years, respectively).

The continuous variables were expressed as the mean ± standard deviation (SD), while frequency distributions were used for the qualitative variables. Logistic regression analysis was used to explore the sociodemographic, functional and clinical factors independently associated with the presence of CIND/dementia. The enter method was used in a first step to include the adjusting variable (patient age and sex), followed in a second step by the stepwise method in application to the rest of the independent variables: years of education, educational level, Katz index, living with partner, restlessness, anxiety or depression, sleeping problems or insomnia, diabetes and Charlson comorbidity index. The dependent variable was cognitive impairment (CI)(code 0: no CI; code 1: presence of CI), while in the case of the independent variables the reference groups were male sex, illiteracy, absence of diabetes, no insomnia, no depression, and living without a partner. Patient age, the Katz index, years of education and the Charlson comorbidity index were taken as continuous variables. The data were analyzed using the SPSS/PC+ version 18.0 statistical package (SPSS Inc., Chicago, IL, USA).

## Results

**Figure 2:**
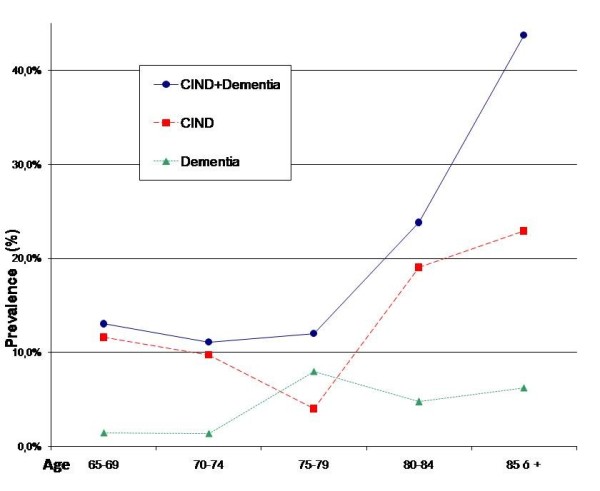
**Crude, age-adjusted and age- and sex-adjusted prevalence of CIND and dementia**.

Table 2 shows the raw, age-adjusted and age-and sex-adjusted prevalence of CIND (48 participants, 14.7%) and dementia (14 participants, 4.3%). In total, 62 of the 327 participants studied (19.0%) suffered from CI (CIND or dementia). On standardizing for age, the prevalence decreased similarly in men and women - the estimated adjusted prevalence being 16.1% (95%CI: 11.6-20.6), versus 14.9% when adjusted for age and sex. The prevalence of CIND and dementia increased with age (p < 0.001), and women were seen to predominate among the individuals with CIND (79.16%) and in those with dementia (71.42%) (Figure 2). The 14 patients classified as presenting dementia had a mean age of 79.35 years (SD = 7.33), and in 8 cases (57.14%) the cause corresponded to Alzheimer's disease (AD), in 3 cases (21.43%) to probable vascular disorders, and in 3 cases (21.43%) to a mixture of factors.

**Table 2:** Crude, age-adjusted, and age- and sex-adjusted prevalence of dementia and cognitive impairment (CI).

	Crude			Age-adjusted^a^		Age, sex ajusted^a^		No. individuals^b^	
	**PP**	**PE**	**95 CI**	**PP**	**95 CI**	**PP**	**95 CI**	**Count**	**95 CI**

**CI (Dementia+ CIND)**									

**Male**	14/116	12,1	6,1 18,0	10,7	4,6-16,9				

**Female**	48/211	22,7	17,1-28,4	19,1	12,9-25,2				

**Both**	62/327	19,0	14,7-23,2	16,1	11,6-20,6	14,9	10,6-19,2	51	36-65

**Age-group**									

**65-69**	9/69	13,0	5,1-21,0						

**70-74**	8/72	11,1	3,9-18,4						

**75-79**	9/75	12,0	4,6-19,4						

**80-84**	15/63	23,8	13,3-34,3						

**≥ 85**	21/48	43,8	29,7-57,8						

**CIND**									

**Male**	10/116	8,6	3,5 - 13,7	8,1	2,5-13,6				

**Female**	38/211	18,0	12,8 - 23,2	15,1	9,6-20,6				

**Both**	48/327	14,7	10,8 - 18,5	11,4	7,5-15,4	11,6	7,7-15,5	39	26-53

**Age-group**									

**65-69**	8/69	11,6	4,0-19,1						

**70-74**	7/72	9,7	2,9-16,6						

**75-79**	3/75	4,0	0,0-8,4						

**80-84**	12/63	19,0	9,4-28,7						

**≥ 85**	11/48	22,9	11,0-34,8						

**Dementia**									

**Male**	4/116	3,4	0,1 - 6,8	2,7	0,0 - 5,3				

**Female**	10/211	4,7	1,9 - 7,6	4,0	1,3 - 6,8				

**Both**	14/327	4,3	2,1 - 6,5	3,4	1,5 - 5,3	3,3	1,4 - 5,2	11	5-18

**Age-group**									

**65-69**	1/69	1,4	0,0 - 4,3						

**70-74**	1/72	1,4	0,0 - 4,1						

**75-79**	6/75	8,0	1,9 - 14,1						

**80-84**	3/63	4,8	0,0 - 10,0						

**≥ 85**	3/48	6,3	0,0 - 13,1						

**^a^**Age standardization weights European Standard: 36.4, 27.3, 18.2, 9.1 and 9.1 for 65-69, 70-74, 75-79, 80-84 and ≥85, respectively; sex standardization weight: 0.50 (sources: http://www.wmpho.org.uk/localprofiles/metadata.aspx?id=META_EUROSTD) [26];

**^b^**Estimated number of subjects in hundreds with de disease based on age- and sex-adjusted prevalence (Salamanca h ≥65 years 2009 estimated population: 34.020h. (source: Castilla and León Regional Health Service lists. CIND: cognitive impairment not dementia; CI, confidence interval; PE, prevalence point-estimate; PP, crude prevalence ratio (cases divided by population).

**Figure 3:**
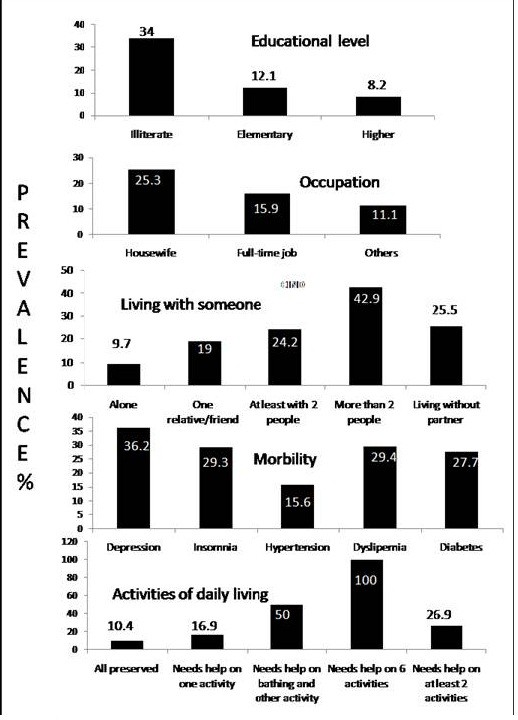
**Prevalence of CIND, CI or dementia according to level of studies, the features functional, clinical and neuropsychological participants**.

Table 3 shows the characteristics of the 327 DERIVA study participants, stratified by cognitive status. Mean age was lower in the normal cognition group, while educational level was higher. The patients with CI had lower Charlson comorbidity scores. Figure 3 shows the prevalence of CIND according to educational level and the functional clinical and neuropsychological characteristics of the participants. The prevalence of CI among the illiterate participants was 34%, versus 25.5% among the patients living without a partner. The prevalence of CI among the individuals with depression, insomnia, hypercholesterolemia or diabetes was over 27%. In turn, 10.4% of the participants who were independent for their activities of daily living (ADLs) as assessed with the Katz index suffered from CI, versus 26.9% of the participants found to be dependent for two or more ADLs.

**Table 3:** Sociodemographic, functional, clinical and neuropsychological characteristics of the participants according to cognitive impairment (CI).

	Healthy subjects N (%)	Subjects with CI N (%)	p
**Number of subjects = 327**	265(81.0)	62 (19.0)	

**Age**^#^	75.46 ± 6.83	80.15 ± 8.18	<0.001

Male	75.26 ± 6.52	80.00 ± 7.22	0.013

Female	75.58 ± 7.04	80.19 ± 8.50	<0.001

**Sex**, n (%)			0.018

Male	102 (87.90)	14 (12.1)	

Female	163 (77.30)	48 (22.7)	

**Years of education**^#^	8.77 ± 2.77	6.93 ± 2.81	<0.001

**Educational level**, n (%)			<0.001

Iliterate	68 (66.0)	35 (34.00)	

Primariy-Secondary education	152 (87.90)	21 (12.1)	

Higher education	45 (91.8)	4 (8.2)	

**Regular occupation in his/her life**, n (%)			0.137

Housewife	68 (74.70)	23 (25.30)	

Full-time job	174 (84.10)	33 (15.90)	

Others	8 (88.90)	1 (11.1)	

**Living with his/her partner**, n (%)	158 (86.30)	25 (13.7)	0.007

**Number of alive children **(Mean ± SD)	2.41 ± 1.87	2.09 ± 1.99	0.265

**Living with someone**, n (%)			0.065

Alone	56 (90.30)	6 (9.70)	

With one relative/friend	149 (81.0)	35 (19.00)	

With at least with 2 people	47 (75.80)	15 (24.20)	

With more than 2 people	4 (57.10)	3 (42.90)	

**Functionals**			

**Activities of daily living (Katz Index):**			**<0.001**

All preserved	120 (45.30)	14 (22.60)	

Needs help on one activity	49 (18.10)	10 (16.10)	

Needs help on bathing and other activity	1 (0.40)	1 (1.60)	

Needs help on 6 activities	0 (0.00)	2 (3.20)	

Needs help on at least 2 activities, but not classifiable on previous categories	95 (35.80)	35 (56.50)	

**Clinicals**			

Depression	30 (11.30)	17 (27.40)	0.001

Insomnia	41 (15.50)	17 (27.40)	0.027

Taking medicine for depression	28 (10.6)	17 (27.40)	0.001

Taking medicine for insomnia	38 (14.30)	15 (24.20)	0.058

Hypertension	119 (45.20)	22 (35.50)	0.163

Dyslipidemia	12 (4.50)	5 (8.10)	0.259

Diabetes Mellitus	47 (17.70)	18 (29.90)	0.045

Charlson comorbidity Index (by age)	3.90 ± 1.46	4.50 ± 1.23	0.004

**Neuropsychologicals**			

MMSE (0-30)^# ^	27.30 ± 2.05	19.12 ± 4.74	<0.001

MMSE			<0.001

0-13	0 (0.00)	5 (8.10)	

14-23	3 (1.10)	43 (69.40)	

24-28	162 (61.10)	3 (4.80)	

>28	81 (30.60)	0 (0.00)	

Benton temporal orientation test	5.16 ± 15.96	38.00 ± 35.66	<0.001

Clock drawing test#	5.97 ± 1.52	3.91 ± 2.35	<0.001

Category Fluency#	15.90 ± 5.04	9.45 ± 4.75	<0.001

7 Minute Screen (Total)#	48.82 ± 14.48	67.38 ± 31.87	<0.001

CI: cognitive impairment (CIND or dementia); MMSE: Minimental State Examination.

#: Mean ± Standard deviation

p: Statistically significant differences between the two groups

MMSE: Mini-Mental State Examination score.

Significant predictor factors were found with the multivariate analyses: age (OR = 1.08, 95%CI: 1.03-1.12), anxiety-depression (OR = 3.47, 95%CI: 1.61-7.51), diabetes (OR = 2.07, 95%CI: 1.02-4.18) and educational level (OR = 0.79, 95%CI: 0.70-0.90) (Table 4).

**Table 4:** Variables associated with presenting cognitive impairment (CI): OR and 95 confidence intervals for total CIND/dementia vs. Controls/Healthy.

	B	OR (95% CI)	P
Age	0.07	1.08 (1.03-1.12)	0.00

Gender	0.58	1.79 (0.88-3.64)	0.11

Years of education	-0.24	0.79 (0.70-0.90)	0.00

Depression-anxiety	1.25	3.47 (1.61-7.51)	0.00

Diabetes	0.73	2.07 (1.02-4.18)	0.04

## Discussion

The observed prevalence of 14.7% for CIND and 4.3% for dementia implies a total of 19% of all people aged over 65 with CI in the city of Salamanca in 2009. The age-adjusted and age-and sex-adjusted overall prevalence (CIND or dementia) was 14.9% (95%CI: 10.6-19.2). The prevalence of CI increased with age and decreased with increasing educational level. Although CI was more frequent among women and in people living without a partner, these characteristics were not significantly associated with CI risk. Significant risk factors were found with the multivariate analyses: age, anxiety-depression and diabetes, while years of education proved to be a protective factor.

Few population-based studies of CI have been published in Spain. Adequate comparisons are therefore difficult to make, though our data are within the lower range of other Spanish studies (13.8-35.2%) [27-31] and European surveys [1,10,32]. A possible explanation for this relatively low prevalence may be the fact that our study was conducted in an urban setting. Large differences have been found among different Spanish regions. In this regard, Murcia yielded a CIND prevalence of 13.8% in the urban setting, versus 23.3% in the rural zone [27]. Nunes et al. [10], in an urban setting in the north of Portugal, reported a lower prevalence of CI (12.0%), though these authors included a younger age group (55 to 79 years). The raw prevalence of CI found in our study is lower than the 22.2% reported for the United States [24,33] or the 16.8% prevalence of CIND and 8% prevalence of dementia estimated for Canada [12].

In our study we took into account the most up-to-date diagnostic criteria [4,5,24] according to which it is considered that CIND can involve alteration in various higher cognitive functions, and not only in memory. Therefore, we should have obtained higher figures than if we had considered the criteria of Petersen et al [34]. On the other hand, however, the Katz ADL Index is less sensitive for assessing ADLs than for assessing more complex activities.

The literature offers more references to the prevalence of dementia, though consensus in this case is likewise lacking regarding the true figures applicable to Spain. Nevertheless, it has been suggested that the prevalence in this country is lower than in other regions of Europe. Our data point to an adjusted dementia prevalence in the city of Salamanca of 3.3%, which is close to the lowest estimates both in Spain [2,6] and in Europe [32]. Crude dementia prevalence for elders aged 70 years and over range from 5% in Murcia [27] to 17.2% in Pamplona [2].

Recent publications suggest that the number of individuals with CIND in the United States is about 70% higher than the number with dementia. In the 71- to 79-year-old age group, 16% had CIND, whereas an additional 5% had dementia. A similar proportion was found in the recent Mexican Health and Aging Study [35], though the prevalence figures are slightly higher: 6.1% for CIND as against 28.7% for dementia. Another possible reason why these differences cannot be explained is the threshold set, since, as Seshadri et al. [36] pointed out, depending on where the differential threshold is placed, the percentages of CIND and dementia will vary. It should also be borne in mind that when a report is requested from an informant about the person's functional limitations, the prevalence is substantially lower [2,6,11]

It must be taken into account that the study was designed to determine the global prevalence of CI, and that the estimation of dementia prevalence would require a larger sample. These results therefore must be viewed with caution. The study was carried out in a random sample of the population of the city that included people living in homes for the elderly, and since the prevalence of dementia in these institutions is higher than in the community [12] the possible associated bias has been avoided. However, the percentage distribution according to the most likely etiology coincides with the data of most other reports - Alzheimer's disease accounting for over one-half of the cases, followed by vascular dementia.

At present, evaluation of the existing data is the subject of even greater debate than the publication of new data [2,24,34,35,37]. It has been reported that the estimates of probable dementia are higher in surveys than in meta-analyses for the 65-84 year age interval, but similar among individuals aged 85 years and older [38]. In our study we obtained sufficient information in the context of a health survey, including evaluations of patient functionality and clinical and neuropsychological conditions, to determine the cognitive status of each participant [13]. Such information is therefore more relevant than that derived only from the application of a battery of tests. The diagnostic criteria employed are similar to those used in recent epidemiological studies [33,36] with the purpose of obtaining results that can be compared with those collected in other settings, and also of examining the tendencies in disease prevalence in a single district in relation to morbidity among the elderly, healthcare, social support and economic resources. In accordance with the current recommendations for conducting epidemiological studies, on including patients with cognitive impairment, we placed priority on the inclusion of all individuals who possibly presented such impairment - since it was not our main objective to analyze the types or the severity of dementia. In other words, we placed greater emphasis on the use of those evaluating instruments affording greater sensitivity, at the cost of lesser specificity. Those surveys based only on the detection of dementia can underestimate the true incidence of neurodegenerative diseases in their milder stages. Considering all of the above, it is even more striking that the CI prevalences found are among the lowest published to date. There is no consensus regarding which functionality scales and neuropsychological batteries [39] are best suited to use in epidemiological studies in dementia, despite the fact that both elements have classically been used in diagnosing the disease [34]. It is exceptional for prevalence studies to specify how functionality has been evaluated [8], though we agree with Thomas et al. [40], who recommended the incorporation of functional disability data as a complement to studies estimating the prevalence and severity of CI in the community. In our study, functionality was evaluated with the Katz ADL index, which is widely used to evaluate elderly people in an objective manner, and is therefore adequate for establishing comparisons among different populations. Among the participants of the DERIVA study, only 45.30% of those classified as not presenting CI, versus 22.60% of those classified as presenting CI, preserved functionality in all the areas evaluated by the Katz ADL index. Disability is common among people over 65 years of age; as a result, the way in which disability is evaluated exerts a strong influence when it is considered in the diagnosis of CI. Both the Lawton PSMS-IADL and the Katz scales remain widely used in psychosocial intervention research, and are easy to complete. The PSMS-IADL is not as popular as the Katz index, but has an option for patient self-report. The Katz and PSMS-IADL have been used widely in anti-dementia drug studies and in some psychosocial intervention studies in both North America and Europe [41,42]. Although the classical criteria of mild cognitive impairment (MCI) [34] excluded the presence of important functional deficits, the need to revise this approach has recently been suggested [43]- particularly when distinguishing between dementia and CIND, where different types of impairment are included [44]. Difficulties remembering appointments, telephone numbers, family meetings, holidays, medicines or domestic economics, or running businesses or filling out certain documents, can all be useful for suspecting early-stage CI [45]. Although less extensively studied, it is also necessary to consider statistical analyses of the results obtained with the different tests, taking into account that patient problems in obtaining better scores are not always attributable to CI. As an example, it has been described that 11 of the 30 items of the MMSE show high variability due to aspects unrelated to the degree of CI. Therefore, the MMSE would not be recommended as an instrument for use in screening for dementia among patients with Parkinson's disease [46].

### The importance of certain personal factors

Our data coincide with those of practically all studies regarding the increase in CI with advancing age (Figure 2). It has been estimated that between 65 and 85 years of age, the prevalence doubles every 5.2 years, in an exponential manner [47]. However, there is no general agreement regarding prevalence in terms of patient sex. The condition is rarely associated with the male sex [48], while in contrast many studies have associated dementia with the female sex [2,29,31,49,50], since women predominate in descriptive studies, and a correlation is found in the bivariate analyses, in accordance with our own observations. However, on considering other variables such as age, educational level or comorbidity, this relationship disappears. Our data would support the idea that dementia is not associated with women [1,25,27,33,47]. In coincidence with our own study, many authors [7,33,51] have described an inverse relationship between the prevalence of CI and a lower educational level. Thus, it has been suggested that these differences could be related to certain biological mechanisms that would be responsible for this association [8,27,29,52,53]. However, a recent epidemiological study based on 875 necropsies revealed no protective effect of the years of education received in early stages in life in relation to the accumulation of neurodegenerative or vascular pathologies of the brain [54]. Even so, it has been suggested that when the disease affects people with higher educational level, the manifestations are milder, being mitigated by a greater coping capacity - thereby postponing their consequences for a period of time. It remains to be clarified whether educational levels developed in later periods in life may or may not affect the development of dementia. In an attempt to offer information on this issue, we analyzed the type of regular professional activity of the DERIVA study patients before retirement, though no relationships were found. Perhaps a different classification of activity, analyzing those which may contribute most to intellectual development, could help identify a relationship. Regarding the family situation, we found that those participants living with their partner showed a 13.7% lower prevalence of CI than those living without a partner (p = 0.007). This is in agreement with the observations of Helmer [55], who found single individuals to have a greater risk of suffering dementia than married people. In our case there were no differences related to the fact of living alone or with one or more people, in terms of the prevalence of CI. As ours is a cross-sectional study, however, neither of these characteristics offers information for clarifying whether the current situation is a consequence of or a risk factor for the development of CI.

Many chronic illnesses can be found in elderly people. The Charlson score was greater among the individuals with CI (p = 0.004), though the multivariate analysis did not find it to behave as a risk factor for CI. The association between diabetes (p = 0.045) and dementia has already been described in classic studies [52], though it must be mentioned that while arterial hypertension is accepted as being more closely associated with stroke [31,51], diabetes is the disease found to be associated with CI in the logistic regression analyses. The association between CI and diabetes, as well as the absence of an association with arterial hypertension and hypercholesterolemia, coincide with the findings in another Spanish region [56]. It appears that brain damage would be related to vascular mechanisms [33,37], and since the prevalence of these cardiovascular diseases is not homogeneous in all regions, they should be considered in CI prevalence studies with a view to establishing comparisons. People with CI more often have sleeping problems (p = 0.027) and anxiety-depression (p < 0.001), though only the latter was seen to behave as a CI risk factor in the multivariate analysis. In coincidence with Johansson et al. [57], we found an association between psychological stress and CI, though in the study by the mentioned authors this association was detected in middle-aged women.

Among the limitations of our study, mention must be made of the lack of consensus on the precise criteria involved in making comparisons between different epidemiological studies. Another common and important limitation in studies of this kind is the possible loss of the more seriously deteriorated individuals, since it has been shown that those people who refuse to participate are more likely to have more seriously impaired cognitive function [58] - a situation which may introduce bias and alter the prevalence data obtained. A further limitation of the study is that since the evaluations were carried out by four different psychologists, inter-observer reliability may be affected. However, each evaluation was followed by an appraisal of the interview with a view to tests correction and the reduction of possible bias. Among the strengths of the study, we should mention the inclusion of a representative sample from the city of Salamanca, as well the proposal to incorporate functional evaluation and the clinical processes influencing the prevalence of CI [5]. The study cohort would permit a longitudinal evaluation of CIND, and the long-term results may contribute to identifying the characteristics of those individuals who develop dementia.

## Conclusions

The observed raw prevalence of CI was 19% (14.9% after adjusting for age and sex), and corresponds to the lower range of the prevalence estimated at both national and international level. Older age and the presence of diabetes and anxiety-depression increased the risk of CI, while higher educational level reduced the risk.

## List of abbreviations

AD: Alzheimer's disease; BADL: basic activities of daily living; CI: Cognitive Impairment; CIND: Cognitive impairment - no dementia.

## Competing interests

The authors declare that they have no competing interests.

## Authors' contributions

Conception of the idea for the study: ERS, RGG, LGO, MGM and MVP. Development of the protocol and organization: ERS, SMS, CPA and AEH. Participated in the design of the study and performed the statistical analysis: ERS, SMS, CPA and AEH. Writing of the manuscript: ERS, SMS CPA and LGO. All the authors have read the draft critically, so as to make contributions, and have approved the final text. The project was developed by the Primary Care Research Unit at La Alamedilla Health Centre, Salamanca. Spain.

## Pre-publication history

The pre-publication history for this paper can be accessed here:

http://www.biomedcentral.com/1471-2377/11/147/prepub
